# Beyond binary: Analyzing closed‐source data to compare specific roles and behaviors within violent and nonviolent terrorist involvement

**DOI:** 10.1111/1556-4029.15648

**Published:** 2024-11-07

**Authors:** Amber Seaward, Zoe Marchment, Caitlin Clemmow, Frank Farnham, Richard Taylor, Luc Taperell, Sara Henley, Sara Boulter, Karen Townend, Paul Gill

**Affiliations:** ^1^ Department of Security and Crime Science University College London London UK; ^2^ North London Forensic Services Enfield UK; ^3^ UCL Jill Dando Institute of Security and Crime Science London UK

**Keywords:** risk and protective factors, risk assessment, terrorism, terrorist roles and behaviors, threat assessment, violent and nonviolent terrorist behaviors, violent extremism

## Abstract

Increasingly, studies compare risk and protective factors for involvement in violent and nonviolent terrorist behaviors. This exploratory study investigates whether this distinction is sufficient, or whether it should be disaggregated further into more granular terrorist roles and behaviors. Using data on 404 referrals to a UK countering violent extremism Prevent hub specializing in mental health and associated needs, we compare violent and nonviolent referrals, and then more specific behaviors (vulnerability, proactive extremism, foreign fighting, and violence planning). Bivariate and multivariate analyses show there is value in disaggregating beyond the binary violence versus nonviolence distinction, as more (and more detailed) relationships emerged when using the disaggregated set of behaviors. While gender did not differentiate violent and nonviolent referrals, women were more likely to be referred for radicalization vulnerability or potential foreign fighting. Extreme right‐wing and extreme Islamist referrals were no more or less violent overall, but Islamist referrals were disproportionately referred for both the most and least violent behaviors. Personality and developmental disorders were associated with violence, and disaggregated behaviors provided detailed insight into the drivers of these associations. These exploratory findings, while interesting, likely do not generalize beyond our specific sample. Instead, this study's value lies in demonstrating the utility for both research and, eventually, practice of disaggregating beyond violence and nonviolence. The results demonstrate clear operational implications for threat assessment in the need to include a more refined set of risk factors to aid in assessing risk of more relevant outcomes than terrorist involvement overall.

**Highlights**

- There is value in disaggregating beyond the binary violent and nonviolent terrorism distinction.
- More (and more detailed) relationships emerged when using a disaggregated set of behaviors.
- For research, disaggregated behaviors could be used to better identify terrorism risk factors.
- For threat assessment, disaggregated behaviors are more relevant to outcomes of concern.

## 1 INTRODUCTION

Threat assessment and management is a process of identifying, assessing, and disrupting an individual's mobilization to violent or other harmful activities [1, 2]. Best practice involves using a structured professional judgment (SPJ) approach to combine empirically validated risk factors with clinical judgment [3]. Threat assessment is increasingly used in counterterrorism, due to various policies and practices allowing for early identification, and the prevalence of mental illness and grievances offering intervention opportunities. It is now systematically used in the Dutch Police, Australian fixated threat assessment units, and the United Kingdom's Channel Programme, among other settings [4].

Because threat assessment must be informed by a set of empirically supported risk factors for a specific behavior of concern [5], its application to terrorism faces two broad challenges. First, there are few empirically validated risk factors available [1, 6]. Individuals involved in terrorist behavior are a heterogeneous group in their sociodemographic background, psychological factors, ideological knowledge, belief systems, and behavior [7, 8]. Consensus suggests it is impractical, infeasible, and unethical to develop a “terrorist profile” [9]. Similarly, due to the variety of radicalization pathways, research supports a range of competing, and sometimes mutually incompatible, models of radicalization [9, 10]. As it is not possible to profile either terrorists or the process of becoming a terrorist with sufficient sensitivity and specificity [11], attempting to assess the threat of an individual being involved in terrorism is challenging.

Second, a threat assessment process requires assessing the likelihood of a specific behavior of concern [5, 12]. “Terrorist involvement” may be too broad for this purpose [13, 14]. It lacks specificity. Involvement in this instance could comprise multiple outcomes including ideological support, recruitment, financial facilitation, propaganda, weapons provision, attack planning, and a range of modes of violence (e.g., suicide bombing, vehicular assault, etc.) [3, 12, 15, 16]. Each outcome may feasibly have a different set of risk factors [5]. Some threat assessment cases may seek to target terrorist involvement in the aggregate, one of these roles, a specific phase of radicalization, or use of a specific method [5, 6].

Both problems may result from the literature's tendency to use terrorism as a single dependent variable [8, 17, 18, 19], which fails to account for variability in terrorist outcomes and subcategories of roles and behaviors within that aggregated label [18, 20]. It also represents a bias in analyzing only individuals who are both radicalized *and* involved in terrorism, given radicalization is neither a necessary nor sufficient condition for terrorism [21]. For research, this tendency may obscure important relationships and risk factors hidden within a seemingly heterogeneous group that appears to have no profile [15, 22].

We argue the most operationally relevant empirical research should use a dependent variable that disaggregates specific behaviors, rather than overall terrorist involvement [1, 11, 12, 17, 18]. Resultingly, this paper uses bivariate chi‐squared analysis and multivariate logistic regression models to answer the following research questions:

Are there differences in:

- Basic demographics (age and gender)
- Ideology, or
- Mental health disorders.

Between:

1. Those involved in countering violent extremism (CVE) programs for violent versus nonviolent behaviors, and:
2. Those involved in specific behaviors within this: vulnerability, proactive extremism, foreign fighting, and violence planning?

We analyze referrals to the United Kingdom's southern vulnerability support hub (VSH), one of three VSHs in England and Wales that started as a pilot in 2016. These operate as part of the Prevent strategy within the safeguarding process to identify and respond to vulnerable individuals with unmet mental health or other associated needs [23]. Individuals already involved in CVE programs are referred to this VSH if there is a potential mental health or other complex need, and the VSH comprises a multidisciplinary team of NHS psychologists, psychiatrists, and nurses, alongside police [23]. Individuals are not clinically assessed or treated in‐house [24]. However, VSH staff use formulation‐based approaches to provide early assessment of mental health needs (including where this informs counterterrorism risk), advise on mental health issues, safeguard individuals' mental health, and help put individuals in touch with mental health or other services, including for housing issues and substance abuse [25].

### 1.1 Disaggregating by level of violence

Simply, not all extremists commit extremist violence [26]. Radicalized individuals, even if supportive of terrorism as an objective, may be unwilling or unable to directly involve themselves in violence. Terrorist organizations and their attacks depend on successful completion of numerous roles and activities that are not all directly violent [5, 16, 18]. Horgan et al.'s [13] examination of those convicted of jihadi‐inspired terrorism in the United States identified a range of behaviors including training, financing, management, and attack planning. Most convicts were uninvolved in planning or carrying out attacks. Similarly, Gill and Horgan [17] find at least five distinct but not mutually exclusive role types in the Provisional Irish Republican Army (PIRA): gunman, bomber, facilitatory criminal, bombmaker, and gunrunner.

Numerous studies have attempted to isolate risk factors distinguishing violent from nonviolent extremists [27]. Challacombe and Lucas [28] compare sovereign citizens using the TRAP‐18 threat assessment framework, finding four of 10 distal characteristics (thwarting of occupational goals, ideological framing, personal grievance, and criminal violence) and four of eight proximal warning behaviors (pathway, identification, leakage, and last resort) positively associated with violent criminal acts, with two further proximal behaviors negatively related to violence (novel aggression and energy burst). Similarly, García‐Andrade et al. [29] find the TRAP‐18's total overall score and both proximal and distal subscores predict repeat violent extremism in the mentally ill. However, both misuse or misrepresent the TRAP‐18, designed to merely structure assessment and management, by aggregating total factor scores. Further studies compare between attackers and those who were of concern to CVE agencies but never progressed to attack involvement, to validate the TRAP‐18. Meloy et al. [30] find four warning behaviors (pathway, identification, energy burst, and last resort) and three distal characteristics (ideological framing, changes in thinking and emotion, and creativity and innovation) were more frequent among lethal attackers than those identified as a national security threat, while directly communicated threats and mental disorders were less frequent among attackers. Goodwill and Meloy [31] validate the structure of the TRAP‐18, in their analysis showing distal characteristics' presence in both attackers and those of concern to law enforcement and mental health agencies, but presence and clustering of proximal warning behaviors among only the attackers. Notably, many of these studies that involve nonattackers who were known to agencies are imperfect comparisons, given the risk management or treatment by such agencies may explain the lack of violent action.

Numerous other studies compare violent and nonviolent individual involvement in terrorism or extremism. Scrivens et al. [27] compare online posting behaviors of right‐wing extremists, finding fewer overall posts but more unstable posting frequency in violent users. Knight et al. [14, 22] compare along both violence and lone‐versus‐group actor dimensions finding several psychosocial factors being more present in violent individuals: trauma, rejection, bullying, exposure to extreme violence, and problems of self‐esteem and underachievement. Obaidi et al. [32] found lower openness to experience, emotionality, and altruism personality traits in those with violent versus nonviolent intentions, among samples of Muslims in Europe and Afghanistan as well as former jihadists. Among radicalized individuals and ideologically motivated criminals, those engaging in violence were also more likely to have less social control, more social learning of violence [19, 33], and more perceived loss of significance [34].

### 1.2 Disaggregating further: By role and behavior type

Much less research, however, has empirically delved deeper into risk factors for different roles, particularly with large samples [18, 35, 36]. Gill and Young [18] compared sociodemographic characteristics of Palestinian suicide bombers with US terrorists of multiple roles, finding significant sociodemographic differences. Others compare along empirically derived typologies. Gill and Horgan [17] compare between PIRA roles they identified. For example, comparatively more women were involved in PIRA bomb planting due to their superior ability to conceal bombs in clothing and prams making them preferred candidates for the bomb‐planting role. Similarly, Simcox and Dyer [37] compare within their typology of al‐Qaeda offense types, finding differences in age, citizenship, and religious conversion. Horgan et al. [8] compare across their actor–supporter–facilitator typology in United States–convicted global jihadists, finding differences in citizenship, criminal history type, ideological expressions, and religious conversion.

Furthermore, Kerodal et al. [38] compare financial crimes with homicide among the far right in the United States, finding crucial differences in socio‐political attitudes and beliefs. Perliger et al. [15] find socioeconomic differences among individuals involved in Islamist terrorist organizations across level of violence of activity, training abroad, and speed of progressing through the organization. Those involved in violent roles were more likely to be single, nonimmigrants, and unemployed, supporting their theories of a lack of social and political efficacy driving risk‐taking and therefore violence. Finally, Evans et al. [39] compare between two roles at one extreme of the violence spectrum: frontline and suicide operatives within ISIS foreign fighters. Significant differences found reveal the value of disaggregating even among extremely violent subsets.

### 1.3 Summing up and moving forward

Together, these studies shed light on significant differences between individuals involved in different terrorist behaviors. They find many of the same factors differentiate roles: age, religious conversion, marital status, and citizenship, for example. However, they are subject to several limitations. The first is the range of independent variables used by existing studies. Crucially, they are all limited to comparisons of sociodemographic characteristics. Threat assessment and SPJ approaches require dynamic factors [40], and ideally protective factors [5]. These are observable, actionable, and malleable, and therefore can be used to both identify and manage risk. Ideally, research would therefore incorporate psychological factors and patterns of behavior.

Another limitation is the range of dependent variables used. Many comparisons provide valuable insight into a subset of the range of violent involvement but exclude large sections that are also necessary to plots and organizations. Evans et al. [39], for example, compare only the extreme end of violence (frontline and suicide ISIS operatives). Kerodal et al. [38] compare financial crimes with homicides but include no other roles or positions. Many other papers do include a wider range of behaviors, but they dichotomize this into violence and nonviolence [41] or attackers and nonattackers [15]. Essentially, further research should capture both the wide range of behaviors and compare between more than two groups.

This relates to the issue that while there is much literature exploring the existence of different roles, there is less investigation of the risk factors for these roles [18]. While Horgan et al. [13] identify different roles in their sample, they only compare lone actors to cell members. Similarly, Gill and Young [18] identify disaggregated conventional terrorist roles, but only compare the aggregation to Palestinian suicide bombers.

Many of these limitations are due to data availability. For example, Gill and Young [18] and Perliger et al. [15] mention the high prevalence of missing data as a factor in their restricting attention to only sociodemographic variables. Data availability also restricts generalizability of findings. Snook et al. [41] acknowledge that their context of US Muslim converts is particularly niche and their work should be replicated elsewhere to be generalizable. Gill and Young [18] aimed to avoid being overly context specific and therefore compared Palestinian suicide bombers and US terrorism convictions. While this is valuable for cross‐contextual comparison, it hinders the extent to which findings isolate differences in role types given also the different contexts. As they suggest, future comparisons would ideally be between the “same organization, the same culture, or the same ideology” [18].

These limitations highlight the need for research into those involved in terrorism that: (1) involves more than just sociodemographic independent variables; (2) analyzes the full spectrum of violent and nonviolent roles; and (3) compares within the same context. This study primarily builds upon the latter two issues, by analyzing a closed‐source regional United Kingdom–based dataset of pre‐criminal referrals to a CVE mental health hub. This presents further advantages. Firstly, traditionally studies in this field use open‐source datasets and are thus limited by biases, completeness, and accuracy [7, 10, 42]. This has meaningful impacts on findings. For example, the few closed‐source studies that gain from access to privileged data find higher rates of mental illness than those reliant on open sources. However, these studies are usually restricted to case studies [7]. Therefore, this study is unique in analyzing a large‐N sample, and without the restrictions of open sources. This is a dataset that has not yet been used in public research and was coded and interpreted following discussion with a VSH forensic psychiatrist, a co‐author of this paper.

Secondly, this pre‐crime sample allows inclusion of individuals at a very early stage of radicalization and involvement, rarely found in studies derived from open sources [16]. It does, however, lack the more extreme examples of outright violence that previous studies of terrorist behaviors can include by virtue of examining convictions or open‐source information on attacks [28, 38].

## 2 METHODS

### 2.1 Data

The specific sample of this study is 404 referrals made to the southern VSH in 2019–2020. At the time, the three VSHs offered slightly different services, and the southern VSH was primarily an enhanced liaison, diversion, and consultation service. The hubs were united by a common objective of safeguarding individuals where there is a mental health need to improve outcomes for individuals referred and for the general public through improved risk management.

Ethics approval was granted by the University College London Department of Security and Crime Science Ethics Committee and the European Research Council Executive Agency Research Ethics Committee. This sample excluded referrals where there was missing or insufficient information regarding the behavior that prompted the individual's involvement with CVE programs, as these behaviors are the focus of this study. This applied to 161 cases where the individual was referred for being: an asylum seeker/refugee (accompanied or unaccompanied), suicidal, other, or a missing/unknown reason. Table 1 displays descriptive statistics for basic demographic information of the remaining sample used for analysis.

**TABLE 1:** Demographic descriptive statistics.

	Frequency	Percentage
Age		
Range 9–71, mean = 30.5, SD = 12.3		
25 and under	160	39.6
25–35	125	30.9
36–45	66	16.3
46–55	39	9.7
56 and over	14	3.5
Gender		
Male	373	92.3
Female	31	7.7
Ethnicity		
White, North European	155	38.4
White/Mediterranean, South European	12	3.0
Black	69	17.1
Asian/South Asian	97	24.0
Arab or North African	37	9.2
Unknown	34	8.4
Ideology		
Chaotic (mixed, unstable, unclear)	9	2.2
Extreme right‐wing	94	23.3
International (non‐Islamist)	45	11.1
Irish Loyalist/Dissident republicans	2	0.5
Extreme Islamist	99	24.5
PKK and Kurdish militants	1	0.2
Other religious	2	0.5
School massacre	5	1.2
Single issue	1	0.2
Missing/unknown/no ideology	146	36.1

### 2.2 Measures

#### 2.2.1 Dependent variables: Behaviors

Specific behavior categorizations: the first dependent variable used in this study is the behavior that prompted the individual's involvement in CVE programs, grouped into categories of behaviors and roles comprising terrorist involvement. Because those referred into the service are primarily in the pre‐criminal space, the data do not contain referrals related to those actively involved in preparing acts of terrorism that would constitute a criminal offense. This is discussed in the limitations.

This study categorizes behaviors as follows, as a continuum placed in order of increasing proximity to and involvement in violence:

1. *Vulnerable individuals*: involved in CVE programs because they are potentially vulnerable to radicalization or already radicalized, but have not acted on this violently or otherwise, nor shown any intention to do so.
2. *Proactive extremists:* involved because they show signs or intentions of acting on radicalization to facilitate future (potentially violent) activity, but are not involved *themselves* in outright physical violence or planning/threatening violence.
3. *Foreign fighters:* involved because they are an active participant in an overseas conflict zone or aspiring to travel there. These are individuals who are trained for violence or have the intention of doing so.
4. *Violence planners:* involved because they are recorded as intending to plan attacks, or using/being willing to use outright physical violence.

Violence categorizations: The second dependent variable used is whether the behavior that prompted the individual's involvement in CVE programs was violent or nonviolent. Here, we refer to violence on a direct and individual level, rather than violence being supported as an overall objective. This study largely adopts Knight et al.'s [14] definition of violence as “any act which constituted, or any potential act which, if carried out would constitute, murder, attempted murder, manslaughter, culpable homicide, assault, and/or real injury to another, and/or cause serious and significant structural damage.” As already mentioned, given this sample was limited in excluding convicted terrorists or carried‐out attacks, the violence category also included intentions, plans, or threats for violence. Altogether, included in this study's violence category are actions that either directly lead to violence, or are intended to do so but are either removed from the final step or fail [19, 33]. This aligns with Knight et al. [22] who include both bomb detonating and bomb making, for example. It also means that travel and attempted travel to conflict zones are included in the violent category.

Nonviolent behaviors are therefore those that may indirectly facilitate violence but are not themselves directly violent on the individual level. In this category are those who distribute extremist literature [22], destroy property [33], possess weapons (but without plans to use them) [33], and carry out financial crimes or support for a terrorist organization [33, 34].

Altogether, this led to the categorization of both dependent variables shown in Table 2, again as a continuum of increasing individual involvement in and proximity to violence. In analysis, the violence variable (DV1) was treated as a binary dummy variable (0 = nonviolent and 1 = violent), while the disaggregated specific behavior variable (DV2) was treated as a categorical variable (1 = vulnerable, 2 = proactive extremist, 3 = foreign fighter, and 4 = violence planner). The decision to include active and aspirant travel to conflict zones in the violent category is somewhat subjective, and uncertain due to a lack of further information about what these behaviors mean in the dataset. Therefore, all analyses were repeated with foreign fighters instead in the nonviolent category. This is given in the Supplemental Information, and any changes to results are mentioned in the main text.

**TABLE 2:**
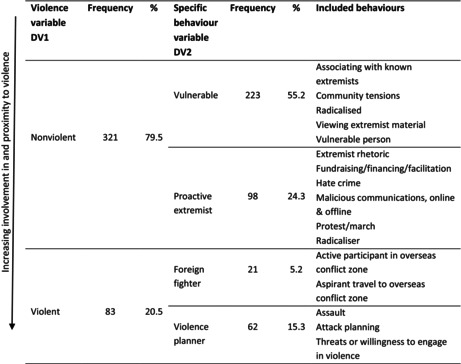
Categorizations for the two dependent variables of violence and specific behavior.

#### 2.2.2 Independent variables

The following independent variables were used in analysis:

Basic demographics: including age as a continuous variable and gender as a binary dummy variable (0 = male, 1 = female). We considered using age as a categorical variable (i.e., under 18, 18–29, and 30+), but given the lack of theory to justify arbitrary cut‐offs and results being heavily dependent on different arbitrary cut‐offs, we decided it was more appropriate to keep age as a continuous variable. Descriptive statistics are summarized in Table 1.

Ideology: Table 1 describes the relative frequencies of the ideologies of individuals included in the sample. For analysis, we compare the two largest identifiable ideologies: extreme right‐wing (XRW) and extreme Islamist, coded as a binary dummy variable (0 = XRW, 1 = Islamist).

Mental health disorders: the dataset includes information on mental health disorders pertaining to individuals referred to the VSH. These are not all or any disorders that the individual is diagnosed with, but instead the disorder that is considered by the VSH clinicians as most relevant to counterterrorism risk for that individual at the time that their case was brought to the attention of the VSH.

Table 3 summarizes relative frequencies of disorders present in this sample. To avoid problems in bivariate and multivariate tests, this study used four disorders with the highest prevalence, where for at least 5% of referrals that disorder was judged as most relevant to counterterrorism risk. These are in bold in Table 3.

**TABLE 3:** Descriptive statistics for mental health diagnosis most relevant to counterterrorism risk.

ICD‐10 code	Frequency	Percentage
Any mental health disorder	277	68.6
Mental disorders due to known physiological conditions, for example dementia	4	1.0
Mental and behavioral disorders due to psychoactive substance misuse, for example NPS, alcohol / opiate dependence	14	3.5
Schizophrenia, Schizotypal, delusional, and other non‐mood psychotic disorders (psychotic disorders)	124	30.7
Mood affective disorders, for example bipolar disorder and major depressive disorder (mood disorders)	31	7.7
Anxiety, dissociative, stress‐related, somatoform, and other nonpsychotic mental disorders, for example PTSD and OCD	19	4.7
Disorders of adult personality and behavior (personality disorders)	32	7.9
Pervasive and specific developmental disorders, for example Autism Spectrum Disorder (developmental disorders)	48	11.9
Behavioral and emotional disorders with onset usually occurring in childhood or adolescence, for example ADHD and conduct disorder	8	2.0
Other/unspecified diagnosis	1	0.2

### 2.3 Analytic procedure and methodology

Bivariate analysis: For each independent variable in turn, we first tested the association between it and the binary dependent variable that indicated whether the individual was involved in CVE programs for violence or nonviolence. The same independent variable was then tested against the disaggregated categorical variable of specific behaviors. This allowed comparison of relationships that emerged when using the binary violence variable, with those that emerged when using the more granular behavioral variable.

Chi‐squared tests measured association between two categorical variables. The chi‐squared statistic was reported, along with its asymptotic two‐tailed significance level. When a significant association was found between two binary variables, odds ratios (ORs) were reported. Cramer's V was used as the effect size to measure the strength of this association, using Pallant [43] as a guide for interpreting this strength as high, medium, or low. Standardized residuals were also examined. Any that exceed ±1.96 provide insight into which relationships are significant in driving the overall chi‐squared relationship at the 5% level [44].

Multivariate analysis: Next, multivariate logistic regressions were conducted, using multiple independent variables. Again, this was firstly done using the binary dependent variable for violence versus nonviolence (binary logistic regression) and then repeated with the categorical variable comparing the four specific behaviors (multinomial logistic regression).

Binary and multinomial logistic regressions were used to model the extent to which independent variables influenced the probability of a referral being violent or involved in a specific behavior, respectively. The independent variable for “any mental health disorder” was excluded, as including this aggregated variable alongside the specific disorders would cause multicollinearity problems. The binary independent variable for ideology was also initially excluded from analysis to prevent the sample size from being reduced from 404 to 193 and the sample being limited to those only involved in XRW or Islamist extremism. All models were then repeated with the inclusion of this variable to ascertain the significance of this ideological comparison alone.

## 3 RESULTS

### 3.1 Bivariate tests of association

Table 4 shows the results of chi‐squared tests of association between each independent variable and both dependent variables.

**TABLE 4:** Test statistics from bivariate chi‐squared analysis.

Independent variable	Violence variable DV1	Specific behavior variable DV2
Gender^a^	0.085	10.567*, ^c^
Age^a^	0.381^d^	0.491^e^
Ideology^b^	0.696	11.786**
Any mental health disorder^a^	0.308	1.118
Psychotic disorders^a^	0.437	2.417
Mood disorders^a^	0.401	1.525^c^
Personality disorders^a^	6.120*	8.164*, ^c^
Developmental disorders^a^	5.458*	6.222

^a^ *N* = 404

^b^ *N* = 193.

^c^ Fisher–Freeman–Halton exact test.

^d^ *t*‐test.

^e^ ANOVA.

* *p* < 0.05.

** *p* < 0.01.

#### 3.1.1 Basic demographics

Gender: overall, women are underrepresented in the sample (7.7%), and just as likely to hold violent (8.4%) and nonviolent roles (7.5%) on the aggregate (*χ* ^2^ (1, *N* = 404) = 0.085, *p* = 0.770). However, women were more likely to be referred for certain specific behaviors (Fisher–Freeman–Halton test, *p* = 0.011). For example, women comprised only 2.0% of proactive extremists, but 19.0% of foreign fighters. The effect size was low (Cramer's *V* = 0.161) meaning this is a weak relationship. Examination of residuals showed this relationship was mainly driven by women being disproportionately unlikely to be proactive extremists compared to men (standardized residual = −2.0). That is, there were fewer female proactive extremists in the sample than would be expected if referrals were distributed randomly across gender and behavior.

Age: As age was the only continuous independent variable, different bivariate tests were employed. The Levene statistic confirmed equal variances, allowing the ANOVA test to be used (Levene statistic = 0.840, *p* = 0.473). Age was unrelated to both violence (*t* = 0.381, *p* = 0.704) and more granular behaviors (*F* = 0.491, *p* = 0.689).

#### 3.1.2 Ideology

XRW and Islamist referrals were no more or less violent (20.2% and 25.3%, respectively (*χ* ^2^ (1, *N* = 193) = 0.696, *p* = 0.404)). However, there is an association with specific behaviors (*χ* ^2^ (3, *N* = 193) = 11.786, *p* = 0.008). For example, XRW comprised 40.0% of vulnerable referrals, but 67.9% of proactive extremists. The effect size was statistically significant (Cramer's *V* = 0.247, *p* = 0.008) with a medium‐level strength of association. Examination of residuals showed this overall relationship was mainly driven by XRW referrals being disproportionately likely (and Islamist referrals disproportionately unlikely) to be proactive extremists (standardized residuals 2.1 and − 2.0, respectively).

#### 3.1.3 Mental health diagnosis

Any mental health disorder: for mental health disorders, firstly those referrals with any mental health disorder considered relevant to counterterrorism risk were compared to those without. For both violent and nonviolent categories, around 70% had a diagnosis relevant to counterterrorism risk. There was no relationship between this variable and violence (*χ* ^2^ (1, *N* = 404) = 0.308, *p* = 0.579), nor specific behaviors (*χ* ^2^ (3, *N* = 404) = 1.118, *p* = 0.773).

However, this can be broken down into the most prevalent specific mental health disorders.

Psychotic disorders: As shown in descriptive statistics, there was a high prevalence of psychotic disorders in this sample. This implies a significant role played by psychotic disorders in willingness or capacity for concerning behaviors in the VSH's caseload. However, there was no relationship between psychotic disorders being the most relevant and violence (*χ* ^2^ (1, *N* = 404) = 0.437, *p* = 0.509). Similarly, there was no relationship with specific behaviors (*χ* ^2^ (3, *N* = 404) = 2.417, *p* = 0.491). Psychotic disorders being the diagnosis most relevant to counterterrorism risk was independent of the behavior or level of violence that initiated the referral.

Mood disorders: Similar dynamics apply to mood disorders. While in 7.7% of referrals mood disorders were considered relevant to counterterrorism risk, there was no relationship between this and violence (*χ* ^2^ (1, *N* = 404) = 0.401, *p* = 0.527), nor specific behaviors (*p* = 0.701). Mood disorders being the diagnosis most relevant to counterterrorism risk was also independent of the behavior or level of violence that initiated the referral.

Personality disorders: while only 6.2% of nonviolent referrals had a personality disorder as the most significant to counterterrorism risk, 14.5% did for violent referrals (*χ* ^2^ (1, *N* = 404) = 6.120, *p* = 0.013), although this difference had a small effect size (Cramer's *V* = 0.123, p = 0.013). An OR of 2.54 reflects the odds of being referred for violent behavior for those with personality disorders as a significant diagnosis to counterterrorism risk being over twice as high as for those without. Relevant personality disorders also differentiated specific behaviors (Fisher–Freeman–Halton test, *p* = 0.034), although with a weak effect size (Cramer's *V* = 0.157, *p* = 0.019). For example, 17.7% of violence planners had relevant personality disorders, compared to only 4.8% of foreign fighters. Examination of residuals showed this overall relationship was mainly driven by those with counterterrorism‐relevant personality disorders being disproportionately likely to be violence planners (standardized residual = 2.7).

Developmental disorders: Developmental disorders were related to violence, as while 19.3% of violent referrals had developmental disorders, only 10.0% of nonviolent referrals did (*χ* ^2^ (1, *N* = 404) = 5.458, *p* = 0.019). Again, this was a weak relationship (Cramer's *V* = 0.116, *p* = 0.019). Similar to personality disorders, the OR of 2.16 reflects the odds of being referred for a violent behavior being over twice as high for those with developmental disorders as the most significant diagnosis to counterterrorism risk. However, developmental disorders being most relevant to counterterrorism risk was *independent* of the specific behavior (*χ* ^2^ (3, *N* = 404) = 6.222, *p* = 0.101).

For bivariate analysis, this was the only result that was changed when foreign fighters were included in the nonviolent category as opposed to the violent category. Here, there was no relationship between developmental disorders and violence (*χ* ^2^ (1, *N* = 404) = 2.403, *p* = 0.121).

### 3.2 Multivariate logistic regressions

Multivariate logistic regression models then explored whether independent variables have power to predict the likelihood of being involved in violence or involved in a specific behavior.

#### 3.2.1 Binary regression: Predicting violence versus nonviolence

Mirroring the process for bivariate analysis, the first regression uses the binary dependent variable of violence and nonviolence. Table 5 shows the output of a binary logistic regression of the factors affecting the likelihood of being referred for a violent behavior compared with a nonviolent behavior (as the reference category). Independent variables included in the model as stated in the methods section were all variables used in bivariate analysis, excluding the binary ideology variable and the variable indicating presence of any mental health disorder relevant to counterterrorism risk.

**TABLE 5:** Factors influencing the probability of being referred for a violent versus nonviolent behavior.

Included variable	B (SE)	95% confidence interval for odds ratio		
		Lower	Odds ratio (Exp(B))	Upper
Constant	−1.772 (0.412)***		0.170	
Age	0.003 (0.011)	0.981	1.003	1.025
Gender	0.278 (0.458)	0.538	1.321	3.242
Psychotic disorders	0.182 (0.311)	0.651	1.199	2.208
Mood disorders	−0.061 (0.532)	0.332	0.941	2.670
Personality disorders	1.143 (0.420)**	1.376	3.316	7.148
Developmental disorders	1.018 (0.398)*	1.268	2.767	6.039

*Note*: *N* = 404, *R* ^2^ = 0.031 (Cox & Snell), 0.048 (Nagelkerke). Model *χ* ^2^ (6, *N* = 404) = 12.515, *p* = 0.051.

* *p* < 0.05.

** *p* < 0.01.

*** *p* < 0.001.

Low values of *R* ^2^ reflect the low predictive ability of this model. However, the table shows that having either personality or developmental disorders as most relevant to counterterrorism risk influences the likelihood that the referral was for a violent behavior as opposed to a nonviolent behavior. Using the ORs, the odds of a referral being violent was around three times higher for cases where either diagnosis was most relevant to counterterrorism risk. All other variables were insignificant predictors. This mirrors what was found in bivariate analysis, where the only variables with an association with violence on the aggregate were personality and developmental disorders.

This model was repeated with foreign fighters in the nonviolent category. Personality disorders remained significant, but developmental disorders were no longer significant at the 5% level (*p* = 0.062). Full results are in Supplemental Information Appendix 1.

This model was then rerun including ideology, with the sample size therefore reduced to 193. The OR for ideology was not statistically significant in the model, and significance of other variables was unchanged. Full results are in Supplemental Information Appendix 2. Again, this was repeated with foreign fighters in the nonviolent category. Results here differed in that the coefficient for ideology was statistically significant, and for developmental disorders was not. Full results are in Supplemental Information Appendix 3.

#### 3.2.2 Multinomial logistic regression: Predicting individual behaviors

For multinomial logistic regression, independent variables matched those included in the binary logistic regression, excluding mood disorders due to a zero observed count in a cross‐tabulation with specific behaviors (zero foreign fighters with mood disorders as the most relevant to counterterrorism risk) causing high standard errors. Again, ideology was included in a separate model repeated afterward. For gender and personality disorders, there were > 20% of expected frequencies less than 5, which violates goodness‐of‐fit assumptions in logistic regressions. They were included in the main model as this was a better fit to the data according to a likelihood ratio test, but the model without these variables is given in Supplemental Information Appendix 4.

Regarding the overall fit of the model, low values of *R* ^2^ reflect weak effect sizes. However, the significant result of a likelihood ratio test shows the model explained a significant amount of variability in the data (*χ* ^2^ (15, *N* = 404) = 29.263, *p* = 0.015). Furthermore, the model's expected values were not significantly different from the observed values, according to Pearson (*χ* ^2^ (435, *N* = 404) = 420.055, *p* = 0.688) and Deviance (*χ* ^2^ (435, *N* = 404) = 363.597, *p* = 0.995) goodness‐of‐fit tests.

Regarding each variable's individual contribution to the entire model in predicting all four specific behaviors, likelihood ratio tests show gender (*χ* ^2^ (3, *N* = 404) = 11.683, *p* = 0.009) and personality disorders (*χ* ^2^ (3, *N* = 404) = 8.006, *p* = 0.046) were both significant. All other variables were not significant: age (*χ* ^2^ (3, *N* = 404) = 0.732, *p* = 0.866), psychotic disorders (*χ* ^2^ (3, *N* = 404) = 2.356, *p* = 0.502), and developmental disorders (*χ* ^2^ (3, *N* = 404) = 6.984, *p* = 0.072). This mirrors what was found in bivariate analysis, where only gender and personality disorders (and ideology) were associated with the variable of specific behaviors.

Table 6 displays the output of the multinomial logistic regression model of factors influencing the likelihood of being referred for each specific behavior in relation to another specific behavior. Here, the coefficients on several variables are statistically significant, meaning they distinguish two specific behaviors from each other (in bold in the table).

**TABLE 6:** Factors influencing the probability of being referred for specific behaviors.

Included variable	*B* (SE)	95% confidence interval for odds ratio		
		Lower	Odds ratio (Exp(B))	Upper
Proactive extremist versus vulnerable				
Constant	−2.426 (1.080)*			
Age	0.003 (0.010)	0.983	1.004	1.024
Gender	−1.666 (0.751)*	0.043	0.189	0.889
Psychotic disorders	−0.202 (0.280)	0.472	0.817	1.414
Personality disorders	0.136 (0.502)	0.428	1.147	3.067
Developmental disorders	0.110 (0.431)	0.479	1.116	2.604
Foreign fighter versus vulnerable				
Constant	0.721 (1.674)			
Age	−0.011 (0.023)	0.946	0.989	1.035
Gender	0.993 (0.621)	0.799	2.703	9.091
Psychotic disorders	0.674 (0.545)	0.673	1.961	5.714
Personality disorders	0.333 (1.116)	0.156	1.395	12.500
Developmental disorders	1.401 (0.703)*	1.024	4.065	16.129
Violence planner versus vulnerable				
Constant	−0.198 (1.048)			
Age	0.008 (0.013)	0.983	1.008	1.033
Gender	−0.664 (0.644)	0.146	0.515	1.821
Psychotic disorders	−0.071 (0.361)	0.459	0.931	1.887
Personality disorders	1.322 (0.468)**	1.499	3.745	9.434
Developmental disorders	0.939 (0.468)*	1.022	2.558	6.410
Proactive extremist versus violence planner				
Constant	−2.228 (1.330)			
Age	−0.004 (0.014)	0.969	0.996	1.023
Gender	−1.003 (0.935)	0.059	0.367	2.294
Psychotic disorders	−0.131 (0.403)	0.398	0.877	1.934
Personality disorders	−1.186 (0.544)*	0.105	0.306	0.887
Developmental disorders	−0.829 (0.528)	0.155	0.437	1.229
Foreign fighter versus violence planner				
Constant	0.919 (1.846)			
Age	−0.018 (0.025)	0.935	0.982	1.031
Gender	1.657 (0.834)*	1.022	5.236	27.027
Psychotic disorders	0.745 (0.621)	0.624	2.105	7.092
Personality disorders	0.989 (1.140)	0.040	0.372	3.472
Developmental disorders	0.463 (0.768)	0.352	1.587	7.143
Foreign fighter versus proactive extremist				
Constant	3.147 (1.876)			
Age	−0.014 (0.024)	0.941	0.986	1.034
Gender	2.660 (0.922)**	2.347	14.286	90.909
Psychotic disorders	0.876 (0.579)	0.772	2.398	7.463
Personality disorders	0.197 (1.157)	0.126	1.217	11.765
Developmental disorders	1.291 (0.748)	0.839	3.636	15.873

*Note*: *N* = 404, *R* ^2^ = 0.070 (Cox & Snell), 0.078 (Nagelkerke). Likelihood ratio test: *χ* ^2^ (15, *N* = 404) = 29.263, *p* = 0.015.

* *p* < 0.05.

** *p* < 0.01.

Gender significantly predicted whether a referral was for proactive extremism or vulnerability. Women were significantly less likely to be proactive extremists than vulnerable compared to men, with an OR of 0.189.

Developmental disorders being most significant to counterterrorism risk significantly predicted whether a referral was for foreign fighting or vulnerability. The odds of those with significant developmental disorders being foreign fighters as opposed to vulnerable individuals were over four times higher than those without (OR = 4.065).

Personality and developmental disorders both significantly predicted whether a referral was a violence planner or a vulnerable person. For those with personality (OR = 3.745) or developmental disorders (OR = 2.558) as most significant to counterterrorism, the odds of being a violence planner as opposed to vulnerable were around three times higher than for those without the disorder as significant.

Personality disorders also significantly predicted whether a referral was a proactive extremist or violence planner. A referral with personality disorders as relevant to counterterrorism risk was significantly less likely to be a proactive extremist than a violence planner (OR = 0.306).

Finally, gender significantly predicted distinguishing a foreign fighter from both a proactive extremist and a violence planner. Women were significantly more likely than men to be foreign fighters than violence planners (OR = 5.236), or proactive extremists (OR = 14.286).

This model was then repeated including the binary ideology variable, with full results reported in Supplemental Information Appendix 5. Ideology was a significant predictor in the overall model (*χ* ^2^ (3, *N* = 193) = 18.401, *p* < 0.001), again as would be expected from its significant association with specific behaviors in bivariate analysis. Furthermore, ideology significantly distinguished proactive extremists from both vulnerable and violence planners. Islamist individuals were significantly less likely to be proactive extremists than vulnerable, compared to XRW individuals (OR = 0.228). Islamist individuals were also significantly less likely to be proactive extremists than violence planners, compared with XRW individuals (0.135).

## 4 DISCUSSION

The overarching finding from this analysis is that there is value in disaggregating further than violence and nonviolence when examining risk factors associated with terrorism. For gender and ideology, in both bivariate and multivariate analysis, no relationship was found with violence versus nonviolence of the individual's behavior. However, when this behavior was separated into more granular activities, significant relationships with gender and ideology did emerge. For personality and developmental disorders where there was a relationship with violence versus nonviolence, disaggregating into more granular behaviors highlighted the specific details and dynamics that were driving this relationship.

For each independent variable, this study also revealed indications of its power, or lack thereof, in distinguishing between individuals involved in different violent and nonviolent behaviors, as explored below. However, these findings should not be overstated. This is exploratory work, and the inclusion of independent variables was driven only by the availability of variables in the dataset rather than prevailing theory or variables that would be operationally useful as risk factors.

Age was not significantly related to violence or specific behaviors in any analysis carried out, implying younger individuals referred to the VSH were no more violent, capable, or concerning. Previous findings regarding age have been mixed. The majority of studies have linked violence to younger age [14, 17, 37]. Klausen et al. [45], for example, find youth is an important risk factor for violent but not nonviolent involvement in American Islamist terrorism, with foreign fighters the youngest of all. In contrast, Meloy et al. [30] found that nonattackers were significantly younger than attackers. The findings from this study contribute to dispelling the myth that most terrorists are aged 18–23 at early involvement [18].

Men are overwhelmingly overrepresented in all stages of terrorism [6, 46], much like other violent crimes. In this sample of those already of concern to CVE agencies, gender was unrelated to violence. While men were overrepresented in the sample, men and women within the sample were no more likely to be involved with CVE programs due to violent or nonviolent behaviors. However, analysis found that gender was associated with individuals' involvement in specific terrorist behaviors. The nature of these relationships explained why no relationship with violence was found. Gender distinguished involvement *within* the nonviolent (vulnerable vs. proactive extremist) and violent (foreign fighters vs. violence planners) categories, more so than it distinguished *between* these categories. Women were more likely to be involved in CVE programs because they were vulnerable than they were proactive extremists, compared to men. Women were also more likely to be foreign fighters than violence planners or proactive extremists, compared to men. In terrorism and violence literature in general, women are less violent. However, this study implies that when women *are* involved in early stages of terrorism or radicalization, gender does appear to have an important role in determining the type of involvement [17]. Other literature makes the point that, although less likely to be involved, when women are involved they are not necessarily passive and do hold active roles [47, 48], and can be involved in terrorism both through being perpetrators and victims [49]. However, this study's findings only apply to women with a mental health need impacting on their risk, so are not representative of those at risk of involvement overall.

Similar dynamics were discovered for ideology when comparing the two most prevalent ideologies in the sample of XRW and Islamism. Ideology was not significant in distinguishing violent from nonviolent involvement, but it did distinguish specific behaviors. Islamist referrals in the sample were more likely to be either vulnerable or violence planners than proactive extremists, compared to XRW individuals, and were therefore overrepresented at both ends of the violence spectrum. Again, this explains the lack of relationship with violence as a binary measure. This is a unique finding, as most comparisons between ideologies compare on individual characteristics and antecedent behaviors rather than their levels of violence or concern, essentially treating ideology as a dependent rather than independent variable [50].

The role, if any, played by mental health in extremism and terrorism is unclear [51], and claims of causation or a role of mental health in counterterrorism strategies are subject to significant controversy [52]. In this study, the first significant finding is regarding prevalence. By virtue of the nature of this VSH and this dataset, for all referrals in this dataset there was some form of mental health difficulty or complex need. However, in almost a third of cases, psychotic disorders were considered by VSH clinicians to be most relevant to counterterrorism risk. This implies some role played by this disorder in radicalization, or in capability and/or intent to commit or facilitate violence, at least as judged by forensic psychiatrists. This aligns with Corner et al.'s [53] finding that lone actors had a higher prevalence of schizophrenia and delusional disorders than the general population.

However, prevalence in this sample was not necessarily related to the violence of the original behavior that prompted involvement in CVE programs: Indeed, psychotic disorders were unrelated to these behaviors nor their level of violence. Instead, it was personality disorders and developmental disorders that, when considered relevant to counterterrorism risk, were related to violence. Analysis of specific behaviors revealed the drivers of this relationship. For personality disorders, this was largely because those with counterterrorism‐relevant personality disorders were more likely to be violence planners than merely vulnerable or proactive extremists. For developmental disorders, those with the disorder were more likely to be either foreign fighters or violence planners than merely vulnerable. These findings are difficult to compare to other studies given they represent not the existence of the diagnosis but the judgment of clinicians of its relevance to counterterrorism risk. Nevertheless, developmental disorders are found to be relevant in other studies [53], and paranoid personality disorders are overrepresented among violent lone actors [22]. More generally, there is a consensus on association between personality disorders and violence, although causal explanations continue to be explored [54]. In this study's sample—where there is a significant mental health need for all individuals—those with personality disorders may emerge as most violent because they do not involve the impairment in functioning that, for example, those with psychotic disorders (the most prevalent) do. Therefore, in the context of this sample, those with personality disorders may have disproportionate capacity for planning and using violence.

However, much like other large samples investigating mental health, this study cannot provide a causal role for mental illness. These disorders may catalyze involvement or may occur as a result of involvement [55]. Furthermore, the relationship could be explained by comorbidity of the disorders with other relevant factors, such as social isolation [51].

Again, it is worth reiterating that these findings regarding specific factors should not be overstated or overgeneralized. This is exploratory work and findings must be heavily caveated due to limitations in the variables used and uniqueness of the caseload of this specialized hub. From these data, we can only conclude that these factors are related to different behaviors when looking at a group that is already of concern for involvement, and presenting with a mental health need—not that they are linked to involvement in terrorism overall. The value of this study is instead its demonstration of the utility of such behavioral distinctions for future research and, eventually, practice. Future research with more operationally relevant risk factors and more generalizable samples should aim to isolate factors and inform future practice.

### 4.1 Limitations

Limitations of this study originate primarily from the nature of the dataset. First, results must be interpreted with awareness of selection effects involved in the sample. This sample does not represent the overall population of individuals of concern to CVE agencies, but those who have then been referred to a specialist hub in a certain region due to mental health and/or complex needs alongside radicalization concerns. Therefore, prevalence rates and relationships found in this analysis apply only to this specific subsample, and should not be generalized to the general population of radicalized or vulnerable individuals and certainly not to those who go on to commit terrorist offenses. Secondly, the sample is largely (at least 73.9%) pre‐criminal. This means the sample does not include the most violent form of terrorist involvement, where individuals have directly carried out attack plots. It does instead have the benefit over datasets that use convictions or open‐source data of including those at the stage furthest from violence (the “vulnerable” category). However, this also means that the “behaviors” in this dataset are primarily *intentions* or *early warning signs* of these behaviors, rather than the behaviors themselves, given that if these behaviors were carried out they would not have remained in the pre‐crime space. Overall, the relationships and risk factors found are therefore not necessarily for the carrying out of these violent and nonviolent behaviors, but the intentions of doing so [56].

Furthermore, the dataset offered a limited set of variables. Although it would be preferable to determine concrete empirical risk factors between different role and behavior types, the variables available in the dataset were limited and hence this work is exploratory. The study was restricted to using age, gender, ideology, and relevant mental health disorders as independent variables. Factors that should be used when assessing risk [1, 57], and are employed by other studies with more detailed datasets [58] (e.g., [7, 14, 22, 50]), are missing. These include more extensive sociodemographic information (e.g., employment, criminal history, marital status, and education), social relationships, behavioral factors, psychological factors, stressors, emotional factors, grievances, cognitive factors, and attitudes or beliefs. As mentioned in the literature review, factors useful to threat assessment are observable, actionable, and dynamic [40], and ideally protective factors [5, 16]. This study was largely unable to overcome the limitations faced by previous studies of restricting attention to sociodemographic variables [15, 18].

The variables that are included in this analysis are also often lacking in information or difficult to interpret. For example, the breakdowns of the aggregated mental health codings were unavailable, so conclusions can only be drawn about developmental disorders in general, rather than autism spectrum disorder specifically. Similarly, there was little information regarding the behaviors that led to CVE involvement, resulting in uncertainty about the level of violence of the active or aspirant travel to conflict zones category. While most other studies analyzing the relationship between mental health disorders and radicalization use diagnoses or symptoms of those disorders as a variable, this study only has data on individuals' diagnoses of specific disorders that are considered by VSH clinicians to be the most relevant to their counterterrorism risk. This does provide an extra layer of information but hinders interpretation of results.

Other limitations are more theoretical and methodological. Ultimately, being able to differentiate between those who are on a path to attack planning versus ideological recruiting, for example, relies on each individual only ever being on a path to one of these behavioral categories. However, terrorism is a complex process where individuals can have multiple roles over time, or at one point in time [8, 13, 17]. Radicalization and involvement in violence are not as linear or chronological as Table 2 implies [2]. Individuals may steadily progress upward over time toward the most violent on the continuum, at each step overcoming boundaries and making decisions about progressing, continuing, or turning back. They may also only ever hold nonviolent positions, hold multiple roles at once, move backward, or skip steps [1, 11, 13, 59]. For example, for some, foreign fighting is a step on the pathway toward domestic attack planning, but others do not require or use this step [36]. This presents problems for research, as the labeled behavior for each referral in this dataset could be temporary, and is not necessarily the end point of that individual's radicalization or violence development. The continuum should be seen as both a series of potentially temporary states in a wider process *and* a series of individual outcomes. Therefore, there may be value in longitudinal studies of roles and their dynamic, rather than static, risk factors [41]. This does not necessarily negate the value of research into roles for practice. Roles can be seen on a continuum of increasing individual involvement in and proximity to violence, as in Taylor and Horgan [59]. Even with the understanding that these roles for one individual can change over time or be held simultaneously, if we were to know a risk factor for or protective factor against, for example, the more “severe” or “violent” end of the continuum, this could feasibly inform risk management for that individual. Ultimately, these findings demonstrate that both practitioners and researchers should be clear on the outcome of concern in threat assessment beyond mere violence versus nonviolence. With further research, there may emerge predictable pathways or patterns to different concerning behaviors that can improve and supplement professional judgments.

Overall, due to these limitations regarding data availability, interpretation, and theory, this study is exploratory. Rather than informing conclusions about empirically supported risk factors from the limited set of independent variables, the findings should be considered conclusions of a “proof of concept” that it is valuable for research and practice to disaggregate terrorist involvement beyond the violent and nonviolent distinction. Research should use this or a similar disaggregation when considering risk factors, rather than comparing merely to general population base rates or to nonviolent extremism. To reinforce this and overcome this study's limitations, future work should use data with larger sample sizes and more (and more operationally relevant) variables. This would involve significant data collection [18] but is crucial to provide a validated evidence base for risk factors for specific terrorist behaviors. Future research could also consider disaggregating further, for example, comparing not just within the same context but within certain ideologies [18], or organizational types (e.g., group vs. lone actor).

## 5 IMPLICATIONS AND CONCLUSIONS

For policy and research, there are dual benefits to the findings and implications of this study: more *identifiable risk factors* for more *relevant outcome behaviors*.

First, for research, there appear to be patterns and dynamics distinguishing individuals involved in different terrorist behaviors that are obscured when such behaviors are grouped together under violence versus nonviolence or, worse, under the overall “terrorist involvement” label [15]. Perhaps the common conclusions from the literature that terrorists are fundamentally heterogeneous have been due to a failure to sufficiently disaggregate this dependent variable and uncover significant subgroups and relationships [15].

Second, for practice, identification of risk factors from such disaggregation would be useful for threat assessment, as it would allow differentiation of more relevant outcome behaviors than just “terrorist involvement” or even violent terrorist involvement. Ideally, threat assessors would be able to judge the level of concern for an individual planning an attack against that of becoming a foreign fighter, a financial facilitator, or an ideological recruiter, for example [2]. This would aid prioritization of finite resources into prevention areas that require different teams, responses, and levels of urgency [14, 16, 18]. More targeted input and interventions, particularly in mental health‐focused hubs, would also improve the tailored service offered to, and therefore improve outcomes for, individuals referred. Future work should use larger samples and more dynamic and behavioral independent variables that can help to consolidate this distinction for practice.

## FUNDING INFORMATION

This work was supported by the European Research Council (ERC) under the European Union's Horizon 2020 Research and Innovation Programme (Grant Agreement No. 758834).

## CONFLICT OF INTEREST STATEMENT

The authors have no conflicts of interest to declare.

## Supporting information


**Data S1:** Supporting Information.
