# Supplementary material for: Beyond binary: Analyzing closed‐source data to compare specific roles and behaviors within violent and nonviolent terrorist involvement
Source: J Forensic Sci. 2024 Nov 7;70(1):222–36. doi: 10.1111/1556-4029.15648 (PMC11693527; doi:10.1111/1556-4029.15648)
Supplement: Supplementary file 1 — Data S1: Supporting Information. [file JFO-70-222-s001.docx]

Appendix 1: Binary logistic regressions from main text with foreign fighters included in the nonviolent category.

TABLE A1 Factors influencing the probability of being referred for a violent vs. nonviolent behaviour, where foreign fighters are included in the nonviolent category.

|  |  | 95% Confidence interval for odds ratio | | |
| --- | --- | --- | --- | --- |
| Included variable | B (SE) | Lower | Odds Ratio (Exp(B)) | Upper |
| Constant | -2.169 (0.460)*** |  | 0.114 |  |
| Age | 0.007 (0.012) | 0.983 | 1.007 | 1.031 |
| Gender | -0.467 (0.634) | 0.181 | 0.627 | 2.171 |
| Psychotic disorders | -0.007 (0.362) | 0.488 | 0.993 | 2.021 |
| Mood disorders | 0.254 (0.543) | 0.445 | 1.289 | 3.736 |
| **Personality disorders** | **1.298 (0.439)**** | **1.548** | **3.661** | **8.660** |
| Developmental disorders | 0.834 (0.447)^ | 0.959 | 2.302 | 5.528 |

*Note: *p < .05, **p < .01, ***p < .001, N = 404, R^2^ = .031 (Cox & Snell), .053 (Nagelkerke). Model χ^2^ (6, N = 404) = 12.524, p = .051.*

Appendix 2: Binary logistic regression with ideology included as a variable.

TABLE A2 Factors influencing the probability of being referred for a violent vs. nonviolent behaviour (including ideology as independent variable).

|  |  | 95% Confidence interval for odds ratio | | |
| --- | --- | --- | --- | --- |
| Included variable | B (SE) | Lower | Odds Ratio (Exp(B)) | Upper |
| Constant | -2.315 (0.692)*** |  | 0.099 |  |
| Age | 0.013 (0.017) | 0.980 | 1.013 | 1.047 |
| Gender | -0.045 (0.719) | 0.234 | 0.956 | 3.916 |
| Ideology (XRW vs Islamist) | 0.754 (0.420) | 0.934 | 2.126 | 4.840 |
| Psychotic disorders | -0.053 (0.477) | 0.372 | 0.948 | 2.415 |
| Mood disorders | -1.337 (1.081) | 0.032 | 0.263 | 2.185 |
| **Personality disorders** | **1.455 (0.559)**** | **1.432** | **4.285** | **12.822** |
| **Developmental disorders** | **1.306 (0.579)*** | **1.186** | **3.690** | **11.478** |

*Note: *p < .05, **p < .01, ***p < .001, N = 193, R^2^ = .074 (Cox & Snell), .133 (Nagelkerke). Model χ^2^ (7, N = 193) = 14.908, p = .037.*

Appendix 3: Binary logistic regressions from Appendix 2 with foreign fighters included in the nonviolent category.

TABLE A3 Factors influencing the probability of being referred for a violent vs. nonviolent behaviour, where foreign fighters are included in the nonviolent category, including ideology as an independent variable.

|  |  | 95% Confidence interval for odds ratio | | |
| --- | --- | --- | --- | --- |
| Included variable | B (SE) | Lower | Odds Ratio (Exp(B)) | Upper |
| Constant | -3.101 (0.816)*** |  | 0.045 |  |
| Age | 0.024 (0.019) | 0.988 | 1.025 | 1.063 |
| Gender | -1.064 (1.088) | 0.041 | 0.345 | 2.915 |
| **Ideology (XRW vs Islamist)** | **0.979 (0.483)*** | **1.032** | **2.661** | **6.864** |
| Psychotic disorders | -0.425 (0.563) | 0.217 | 0.654 | 1.971 |
| Mood disorders | -1.028 (1.094) | 0.042 | 0.358 | 3.056 |
| **Personality disorders** | **1.548 (0.594)**** | **1.468** | **4.702** | **15.059** |
| Developmental disorders | 1.003 (0.684) | 0.713 | 2.726 | 10.421 |

*Note: *p < .05, **p < .01, ***p < .001, N = 193, R^2^ = .074 (Cox & Snell), .127 (Nagelkerke). Model χ^2^ (8, N = 193) = 14.941, p = .037.*

Appendix 4: Multinomial logistic regressions repeated with exclusion of gender and personality disorders.

The model does not explain a significant amount of variability in the data, as shown in a likelihood ratio test (χ^2^ (9, N = 404) = 9.405) p = .401), but does fit the observed data reasonably well, from Pearson (χ^2^ (312, N = 404) = 293.457, p = .768) and Deviance (χ^2^ (312, N = 404) = 292.306, p = .782) goodness of fit tests.

Regarding main effects, no variable significantly predicted specific behaviours: age (χ^2^ (3, N = 404) = 1.239, p = .744), psychotic disorders (χ^2^ (3, N = 404) = 2.577, p = .461), and developmental disorders (χ^2^ (3, N = 404) = 5.102, p = .164).

TABLE A4 Factors influencing the probability of being referred for specific behaviours.

|  |  | 95% confidence interval for odds ratio | | |
| --- | --- | --- | --- | --- |
| Included variable | B (SE) | Lower | Odds ratio (Exp(B)) | Upper |
| **Proactive extremist vs. vulnerable** | | | | |
| Constant | -0.899 |  |  |  |
| Age | 0.004 (0.010) | 0.984 | 1.004 | 1.025 |
| Psychotic disorders | -0.173 (0.272) | 0.493 | 0.841 | 1.435 |
| Developmental disorders | 0.191 (0.427) | 0.524 | 1.211 | 2.793 |
| **Foreign fighter vs. vulnerable** | | | | |
| Constant | -0.640 (0.846) |  |  |  |
| Age | -0.012 (0.023) | 0.944 | 0.988 | 1.034 |
| Psychotic disorders | 0.572 (0.522) | 0.637 | 1.773 | 4.926 |
| Developmental disorders | 1.199 (0.671) | 0.890 | 3.322 | 12.346 |
| **Violence planner vs. vulnerable** | | | | |
| Constant | -1.147 (0.550)* |  |  |  |
| Age | 0.011 (0.012) | 0.987 | 1.011 | 1.035 |
| Psychotic disorders | -0.295 (0.341) | 0.382 | 0.745 | 1.453 |
| Developmental disorders | 0.778 (0.452) | 0.897 | 2.174 | 5.291 |
| **Proactive extremist vs. violence planner** | | | | |
| Constant | 0.249 (0.624) |  |  |  |
| Age | -0.007 (0.014) | 0.967 | 0.993 | 1.020 |
| Psychotic disorders | 0.122 (0.384) | 0.532 | 1.130 | 2.398 |
| Developmental disorders | -0.587 (0.514) | 0.203 | 0.556 | 1.524 |
| **Foreign fighter vs. violence planner** | | | | |
| Constant | 0.508 (0.927) |  |  |  |
| Age | -0.023 (0.025) | 0.931 | 0.978 | 1.026 |
| Psychotic disorders | 0.867 (0.588) | 0.752 | 2.381 | 7.519 |
| Developmental disorders | 0.422 (0.730) | 0.365 | 1.524 | 6.369 |
| **Foreign fighter vs. proactive extremist** | | | | |
| Constant | 0.259 (0.897) |  |  |  |
| Age | -0.016 (0.024) | 0.939 | 0.984 | 1.031 |
| Psychotic disorders | 0.745 (0.551) | 0.716 | 2.105 | 6.211 |
| Developmental disorders | 1.008 (0.715) | 0.675 | 2.740 | 11.111 |

*Note: *p < .05, **p < .01, ***p < .001, N = 404, R^2^ = .023 (Cox & Snell), .026 (Nagelkerke). Likelihood ratio test: χ^2^ (9, N = 404) = 9.405, p = .401.*

The same model was then repeated with the inclusion of the binary variable denoting XRW or Islamist ideology (Table A5). The model explains a significant amount of variability in the data, as shown in a likelihood ratio test (χ^2^ (12, N = 193) = 24.891) p = .015), and fits the observed data reasonably well, from Pearson (χ^2^ (324, N = 193) = 317.315, p = .594) and Deviance (χ^2^ (324, N = 193) = 275.320, p = .977) goodness of fit tests.

Regarding main effects, only ideology significantly predicted specific behaviours (χ^2^ (3, N = 193) = 17.353, p < .001). All other predictors were insignificant: age (χ^2^ (3, N = 193) = 2.272, p = .518), psychotic disorders (χ^2^ (3, N = 193) = 4.509, p = .211), and developmental disorders (χ^2^ (3, N = 193) = 4.802, p = .187).

TABLE A5 Factors influencing the probability of being referred for specific behaviours, including ideology as independent variable.

|  |  | 95% confidence interval for odds ratio | | |
| --- | --- | --- | --- | --- |
| Included variable | B (SE) | Lower | Odds ratio (Exp(B)) | Upper |
| **Proactive extremist vs. vulnerable** | | | | |
| Constant | -1.245 (0.747 |  |  |  |
| Age | -0.003 (0.015) | 0.968 | 0.997 | 1.028 |
| **Ideology (XRW vs Islamist)** | **-1.501 (0.407)***** | **0.100** | **0.223** | **0.979** |
| Psychotic disorders | 0.747 (0.445) | 0.881 | 2.110 | 5.051 |
| Developmental disorders | -0.612 (0.628) | 0.158 | 0.542 | 1.855 |
| **Foreign fighter vs. vulnerable** | | | | |
| Constant | 0.079 (1.168) |  |  |  |
| Age | -0.024 (0.035) | 0.912 | 0.976 | 1.044 |
| Ideology (XRW vs Islamist) | -0.226 (0.688) | 0.207 | 0.797 | 3.067 |
| Psychotic disorders | 0.799 (0.758) | 0.503 | 2.222 | 9.804 |
| Developmental disorders | 1.139 (0.839) | 0.604 | 3.125 | 16.129 |
| **Violence planner vs. vulnerable** | | | | |
| Constant | -1.356 (0.821) |  |  |  |
| Age | 0.021 (0.019) | 0.984 | 1.021 | 1.060 |
| Ideology (XRW vs Islamist) | 0.137 (0.466) | 0.460 | 1.147 | 2.857 |
| Psychotic disorders | -0.254 (0.548) | 0.265 | 0.776 | 2.273 |
| Developmental disorders | 0.613 (0.78) | 0.489 | 1.845 | 6.944 |
| **Proactive extremist vs. violence planner** | | | | |
| Constant | 0.112 (0.929) |  |  |  |
| Age | -0.024 (0.020) | 0.939 | 0.977 | 1.015 |
| **Ideology (XRW vs Islamist)** | **-1.638 (0.531)**** | **0.069** | **0.194** | **0.550** |
| Psychotic disorders | 1.001 (0.608) | 0.826 | 2.717 | 8.929 |
| Developmental disorders | -1.225 (0.777) | 0.064 | 0.294 | 1.348 |
| **Foreign fighter vs. violence planner** | | | | |
| Constant | 1.435 (1.295) |  |  |  |
| Age | -0.045 (0.037) | 0.889 | 0.956 | 1.027 |
| Ideology (XRW vs Islamist) | -0.363 (0.763) | 0.156 | 0.695 | 3.106 |
| Psychotic disorders | 1.053 (0.863) | 0.528 | 2.865 | 15.625 |
| Developmental disorders | 0.527 (0.953) | 0.261 | 1.692 | 10.989 |
| **Foreign fighter vs. proactive extremist** | | | | |
| Constant | 1.323 (1.250) |  |  |  |
| Age | -0.022 (0.035) | 0.913 | 0.978 | 1.048 |
| Ideology (XRW vs Islamist) | 1.275 (0.729) | 0.858 | 3.584 | 14.925 |
| Psychotic disorders | 0.053 (0.797) | 0.221 | 1.054 | 5.025 |
| **Developmental disorders** | **1.751 (0.918)** | **0.953** | **5.747** | **34.483** |

*Note: *p < .05, **p < .01, ***p < .001, N = 193, R^2^ = .121 (Cox & Snell), .133 (Nagelkerke). Likelihood ratio test: χ^2^ (12, N = 193) = 24.891, p = .015.*

Appendix 5: Multinomial logistic regression with inclusion of ideology variable.

The model explains a significant amount of variability in the data, as shown in a likelihood ratio test (χ^2^ (18, N = 193) = 41.306) p = .00), and fits the observed data reasonably well, from Pearson (χ^2^ (378, N = 193) = 378.391, p = .485) and Deviance (χ^2^ (378, N = 193) = 294.912, p = .999) goodness of fit tests.

Regarding main effects, ideology significantly predicted specific behaviours (χ^2^ (3, N = 193) = 18.466, p < .001), as did personality disorders (χ^2^ (3, N = 193) = 9.432, p = .024), and developmental disorders (χ^2^ (3, N = 193) = 7.860, p = .049). Gender (χ^2^ (3, N = 193) = 6.787, p = .079), age (χ^2^ (3, N = 193) = 2.020, p = .568), and psychotic disorders (χ^2^ (3, N = 193) = 2.890, p = .409) were insignificant predictors.

TABLE A6 Factors influencing the probability of being referred for specific behaviours, including ideology as independent variable.

|  |  | 95% confidence interval for odds ratio | | |
| --- | --- | --- | --- | --- |
| Included variable | B (SE) | Lower | Odds ratio (Exp(B)) | Upper |
| **Proactive extremist vs. vulnerable** | | | | |
| Constant | -4.097 (1.623)* |  |  |  |
| Age | -0.001 (0.015) | 0.970 | 0.999 | 1.030 |
| Gender | -1.821 (1.091) | 0.019 | 0.162 | 1.374 |
| **Ideology (XRW vs Islamist)** | **-1.480 (4.17)***** | **0.101** | **0.228** | **0.515** |
| Psychotic disorders | 0.508 (0.455) | 0.681 | 1.661 | 4.049 |
| Personality disorders | -0.766 (0.677) | 0.123 | 0.465 | 1.754 |
| Developmental disorders | -0.834 (0.640) | 0.124 | 0.434 | 1.522 |
| **Foreign fighter vs. vulnerable** | | | | |
| Constant | 1.814 (2.352) |  |  |  |
| Age | -0.026 (0.034) | 0.911 | 0.975 | 1.043 |
| Gender | 0.868 (0.938) | 0.379 | 2.381 | 14.925 |
| Ideology (XRW vs Islamist) | -0.273 (0.718) | 0.187 | 0.762 | 3.106 |
| Psychotic disorders | 1.076 (0.838) | 0.567 | 2.933 | 15.152 |
| Personality disorders | 0.665 (1.242) | 0.170 | 1.946 | 22.222 |
| Developmental disorders | 1.351 (0.896) | 0.666 | 3.861 | 22.222 |
| **Violence planner vs. vulnerable** | | | | |
| Constant | -0.763 (1.720) |  |  |  |
| Age | 0.021 (0.020) | 0.982 | 1.021 | 1.062 |
| Gender | -1.256 (1.095) | 0.033 | 0.285 | 2.433 |
| Ideology (XRW vs Islamist) | 0.523 (0.510) | 0.621 | 1.686 | 4.587 |
| Psychotic disorders | -0.127 (0.583) | 0.281 | 0.880 | 2.762 |
| **Personality disorders** | **1.411 (0.635)*** | **1.181** | **4.098** | **14.286** |
| Developmental disorders | 0.986 (0.722) | 0.651 | 2.681 | 10.989 |
| **Proactive extremist vs. violence planner** | | | | |
| Constant | -3.334 (2.114) |  |  |  |
| Age | -0.021 (0.021) | 0.939 | 0.979 | 1.020 |
| Gender | -0.565 (1.481) | 0.031 | 0.568 | 10.309 |
| **Ideology (XRW vs Islamist)** | **-2.003 (0.576)***** | **0.044** | **0.135** | **0.417** |
| Psychotic disorders | 0.635 (0.641) | 0.538 | 1.887 | 6.623 |
| **Personality disorders** | **-2.177 (0.758)**** | 0.026 | 0.113 | 0.501 |
| **Developmental disorders** | **-1.820 (0.826)*** | **0.032** | **0.162** | **0.818** |
| **Foreign fighter vs. violence planner** | | | | |
| Constant | 2.577 (2.715) |  |  |  |
| Age | -0.046 (0.037) | 0.887 | 0.955 | 1.027 |
| Gender | 2.125 (1.361) | 0.581 | 8.403 | 125.000 |
| Ideology (XRW vs Islamist) | -0.796 (0.811) | 0.092 | 0.451 | 2.212 |
| Psychotic disorders | 1.203 (0.953) | 0.514 | 3.333 | 21.739 |
| Personality disorders | -0.746 (1.285) | 0.038 | 0.474 | 5.882 |
| Developmental disorders | 0.265 (1.036) | 0.189 | 1.441 | 10.989 |
| **Foreign fighter vs. proactive extremist** | | | | |
| Constant | 5.991 (2.672)* |  |  |  |
| Age | -0.025 (0.035) | 0.910 | 0.975 | 1.045 |
| **Gender** | **2.690 (1.360)*** | **1.024** | **14.706** | **200.000** |
| Ideology (XRW vs Islamist) | 1.207 (0.762) | 0.751 | 3.344 | 14.925 |
| Psychotic disorders | 0.568 (0.875) | 0.317 | 1.764 | 9.804 |
| Personality disorders | 1.432 (1.301) | 0.327 | 4.184 | 52.632 |
| **Developmental disorders** | **2.185 (0.982)*** | **1.297** | **8.929** | **62.500** |

*Note: *p < .05, **p < .01, ***p < .001, N = 193, R^2^ = .193 (Cox & Snell), .212 (Nagelkerke). Likelihood ratio test: χ^2^ (18, N = 193) = 41.306, p = .001.*
